# Mr. Mantal, on Mr. Baker's Case

**Published:** 1805-02-01

**Authors:** James Mantal

**Affiliations:** Bromley High Elms


### *To the Editors of the Medical and Physical Journal.*

GENTLEMEN,

HOW much it is to be lamented, that some genius has not yet arisen, who from simple principles, like the immortal Newton, from the simple principle of the general attraction of matter deducing the laws of gravitation, could with equal sagacity develope the laws peculiar to animation, and enlighten mankind with a true theory of diseases! This reflection naturally arises in my mind, while I reflect on the fate which has attended Mr. Baker, of Uxbridge; who, I am afraid, has fallen a victim to the erroneous parts both of the old and new theory of gout.

Mr. Edlin's *pamphlet* professes to give to the world two cases of gout, terminating fatally in consequence of the refrigerating plan of treatment having been adopted. But for obvious reasons, I shall confine my observations to that of Mr. Baker; which, I think, would have been more full and satisfactory, if the author had given us some information respecting the age and usual habits of the patient; whether also his last disorder was the first of the kind he had experienced, or whether his constitutional strength had been impaired by former attacks. For, possessing the knowledge of these particulars, we could assuredly have formed a more correct judgment of the propriety of some parts of the plan of treatment, which terminated in so calamitous a manner. But though the case is not detailed with the precision that I could wish, yet a great deal of valuable instruction may certainly be collected from it; and much light is, I think, thrown on a question, which at present greatly agitates the medical world; namely, how far, or whether in *any* case of gouty inflammation of the extremities, the remedy so strongly recommended by Dr. Kinglake may, in *all* cases indiscriminately, be prudently adopted. I am still of opinion, that judging from all the circumstances which have been communicated respecting Mr. Baker, this very important question may be answered, consonantly to the theory of the disease contained in my last communication, in this manner: *With a due degree of caution*, it may be prudently adopted with every prospect of success.

It will much facilitate our comprehension of this interesting and instructive case to divide it into two periods, that are very distinctly marked; the one previous, the other other subsequent to the fainting-fit. It appears, that from the 22d of July to the morning of the 25th, (four o'clock) the inflammation gradually increased in the feet, and that his appetite during those three days continued unimpaired. Pages 7 and 8. From which circumstances I should infer, that this was a proper time for the application of the new remedy, the inflammation being fixed on the extremities, and the stomach discovering no symptom of torpor, but an evident one of performing its proper function. It was applied; and the event was, what I should expect, that at nine o'clock "the pain was quite gone; he considered himself, in some measure, as well; his pulse at this time was 78." Page 19. Here was every mark of returning health; and had he continued in bed, it is probable that the fainting-fit had not occurred. This I wish to be considered as the first period; for, hitherto, there certainly appears no objection to the remedy that had been employed.

The following observation I make reluctantly; for it reflects on a conduct, which evidently proceeded from the best and purest motives. Was it judicious in Mr. E. to contend with his friend, under circumstances not of complete recovery, but convalescence only, in a long and most interesting argument, page 10 to 19, respecting the propriety of the means which had been employed to subdue the gouty inflammation? Particularly when it is considered, that after a restless and unquiet night, attended with much pain and anxiety, the necessity of recruiting the exhausted fibre by sleep must have been obvious, and should have been pointed out to the patient; and if it could not otherwise be obtained, of procuring it by means of an opiate; instead of agitating his mind, and still further exhausting the animal power, by the necessity imposed on him of combating arguments in the refutation of which he now felt so near, so vital an interest. Though I do not wish to attach much importance to this idea, yet if it had any effect, it must have been a debilitating one; and with some others known, and some probably unknown causes, (amongst the former of which may be reckoned a change of posture from a recumbent to an erect one, so apt to produce vertiginous effects, and sometimes a temporary paralysis of the locomotive muscles in persons of a weak and inirritable habit) must prepare us in some degree for the information which is next conveyed to us. "He soon after got out of bed, and attempted to dress himself; but in the exertion he fell down in a fainting-fit, where he was found by his attendants," page 19.

The reflection, with which I commenced these remarks, recurs to me in this place with double force. I am anxious to discover, (for want of a *true* theory perhaps it cannot be discovered) what was the real efficient cause of the fainting-fit? That it sometimes occurs in regular fits of the gout, where the cure by refrigeration has not been attempted, is well known to those who have experience in this disorder. I am then by no means convinced, that repelled gout was the cause of it in the present instance. Indeed, I am persuaded to the contrary; for we have no experience that repelled gout ever produced such an effect; and I know not by what analogy of reasoning from other phenomena of diseases we are led to such a conclusion. That judicious observer, Dr. Heberden, (page 58 of the Commentaries) tells us, that he has several times known palsies and apoplexies *succeed* immediately to a regular and severe fit. And some cases of a fatal apoplexy are related by Cullen and others to have happened in consequence, as it has been supposed, of *preventing* the gouty inflammation of the extremities in constitutions which had been habituated to it, by the continued use of the Portland powder. And some, perhaps, may fancy that they can discover a faint resemblance between these cases and the fainting-fit, which is the subject of our pressnt consideration. But notwithstanding a suspicion may hence arise, that gout and apoplexy, though with features so dissimilar, are yet of the same family, and have one common parent; the difference between the known means that were employed, as well previously to the effects produced as in the consequences of those effects, precludes all comparison between the respective cases; and therefore, in the present imperfect state of our knowledge of diseases and their causes, we are compelled to consider the fainting-fit as an unexplained event, from which nothing either favourable or unfavourable to the practice of cold ablution can be inferred; until more observations and a longer experience furnish us with similar occurrences under similar circumstances, with which it may be compared. It is evident, that the patient himself had no suspicion that the repulsion of the gout had any connection with the fainting-fit; for he would not afterwards have ventured on a repetition of means, which he conceived had been productive of so alarming an effect.

We cannot, however, be surprised that the system should be so deranged by it, as to exhibit that character of debility which soon appeared in the return of a disorder, order, that could scarcely be considered as totally and effectually removed, even before it happened. We are told, that, "after recovering from the fainting fit, and being put to bed, he took some breakfast," (p. 19); from which circumstance I conclude, that the gout was not yet repelled to the stomach. "A slight inflammatory pain and redness were now observed in the knees," p. 19, evincing that the gout was re-appearing in its true inflammatory form; "but soon subsided by the continued use of cloths dipped in iced water." Here then, in my opinion, was committed that fatal error, to which all the subsequent mischief is to be attributed. The torpor, by this means, produced on the skin, appears to have been immediately followed by those symptoms of a "difficult hurried respiration, cold extremities, quick fluttering intermitting pulse, palpitation of the heart, icy coldness of the stomach, frequent vomitings, and cold sweat," p. 20. Which all demonstrably evinced that the gout was repelled to some of the principal internal vital organs; or, as I think, would be more accurately and intelligibly expressed, that the torpor or inaction of the cutaneous capillary vessels, occasioned by the continued application of iced water over a considerable portion of them, was sympathetically communicated to the membranous parts of those viscera, but principally of the stomach, (already disposed to torpor in consequence of previous debility, by whatever cause induced) whose actions are associated with them. I do not know that this idea of repelled gout is any where to be found directly in authors. It is deduced from combining detached parts of the Darwinian theory.

In this manner a complete destruction of the balance of the moving powers of the system was effected; and if the heart had not possessed sufficient irritability to the stimulus of the blood, to resist in *some* degree the spreading torpor, which was thus alarmingly impressed on the stomach and some contiguous parts, the fatal termination must have occurred instantaneously. Its protraction, and the subsequent events have served to convince me, that the balance, so destroyed, was never again restored to an equilibrium; but from this moment all proceeded irregularly, with some partial remissions and exacerbations, till the complete exhaustion of the principle of animation produced a universal torpor, and destroyed the patient.

It may be useful to make one or two observations on the principles which seemed to direct the medical attendants in treating their patient, during the latter period of

 his his illness. And I must previously assure those gentlemen, that, though I differ from them in opinion, I am actuated by no personal disrespect to them; and, indeed, by no other motive than a wish freely to discuss their theory. The stimulating means that were employed by Mr. Edlin to remove the torpor of the internal parts, appear to me sufficiently judicious, and proved successful. But after its removal, and when the system, possesing accumulated irritability during its torpid state, had acquired unnatural energy of action, evinced by "the hot burning skin, the pulse 96 strokes in a minute, the dry furred tongue, and hurried respiration," (p. 21, 22,) symptoms indicative of an inflammatory condition; Dr. Haworth, who was now joined in attendance with Mr. Edlin, will excuse my presumption in avowing an opinion different from that which he seems to have adopted. The indication in that state of the system, as it appears to me, was to reduce the morbidly increased activity and consequent expenditure of the animal power, by the most prompt and efficacious means; by weakening the undue action of the cutaneous vessels, by exposing them to a current of cool air; and if that had failed, by cold ablution: and, "if the lungs be strongly affected, bleeding is unavoidable and necessary (Heberden's Comment. p. 45,) though we are sure that it is a gouty affection. In all distempers (Com. p. 10,) it is one part of the physician's duty to remove or relieve, as far as can safely be done, the present inconveniences; but the mischief principally to be dreaded in every illness is its tendency to destroy life; and against this the patient is most solicitously to be guarded."

But the theory of Dr. Haworth led him to the conclusion, that as the gout had been repelled, its return to the extremities again must be solicited, p. 21, (which was a good theory a few hours sooner, while the torpor affected the stomach and lungs;) and external and internal stimulation employed for that purpose, p. 22. But the gout, according to my apprehension, had already been driven from the internal parts by the re-action of the system, and had changed its character of gout for one of general inflammation. "The warm regimen, flannel, and cordial diaphoretic draughts," p. 22. What could they do but add fuel to a fire already burning too fiercely; remove to a greater distance every probability of restoring that just equilibrium of action throughout the system, which is essential to health; and by the great expenditure of the principle of of animation, consequent to increased stimulation, accelerate the final catastrophe.

Before I conclude my remarks on this interesting case, I beg leave to observe, that Dr. Kinglake, who frequently challenges the world to produce a single instance of repelled gout consequent to the application of a cold fluid to the inflamed parts, will certainly be expected to reply to, and explain the symptoms asserted by Mr. Edlin to have occurred immediately after its use, on principles which may reconcile them to his theory. Otherwise, I do not see how the alternative is to be avoided, which is contained in Mr. Edlin's concluding paragraph: "Either Dr. Kinglake's theory is wrong, or what I have asserted is untrue,"* p. 24. The alternative, indeed, does not meet with my approbation. It is not expressed with a due degree of modest diffidence; for unless Mr. Edlin's own knowledge be unlimited, he must admit the *possibility*, however little the *probability* may be, of the symptoms being explained on principles that are not inconsistent with the theory. But this province peculiarly belongs to Dr. Kinglake, whose ground of argument I have no wish to pre-occupy; and I suppose it will not be deemed so, by my asking Mr. Edlin, what will become of the alternative, that seems to menace nothing less than destruction to the theory, should Dr. Kinglake be able to prove to our satisfaction, that during the state of insensibility which succeeded the fainting fit, so great an accumulation of the sensorial powers of irritation and association occurred, as to occasion the subsequent inflammatory fever, to which the death of the patient is to be attributed?

I am, &c.

JAMES MANTAL.

*Bromley High Elms, Dec. 11, 1804.*

---

* Could Mr. Edlin complain of Dr. Kinglake, if he chose to consider the whole of Mr. Baker's case as "*fictitious, composed for the purpose*" of discrediting his theory? For these are the expressions used by Mr. Edlin, respecting the cases communicated to the public by Dr. Kinglake, p. 20. I will not bestow upon this seeming incredulity the epithet which I think it deserves. It was an ill compliment to his friend, to suppose that he possessed either so small a portion of candour and liberality of sentiment, or so little judgment, as to listen for a moment to such an insinuation. It is astonishing, that he should avow it to the world.

